# One PMP22/MPZ and Three MFN2/GDAP1 Concomitant Variants Occurred in a Cohort of 189 Chinese Charcot-Marie-Tooth Families

**DOI:** 10.3389/fneur.2021.736704

**Published:** 2022-01-28

**Authors:** Yongzhi Xie, Zhiqiang Lin, Xiaobo Li, Lei Liu, Shunxiang Huang, Huadong Zhao, Binghao Wang, Wanqian Cao, Zhengmao Hu, Jifeng Guo, Lu Shen, Beisha Tang, Ruxu Zhang

**Affiliations:** ^1^Department of Neurology, The Third Xiangya Hospital, Central South University, Changsha, China; ^2^Hunan Key Laboratory of Medical Genetics, Center for Medical Genetics, School of Life Sciences, Central South University, Changsha, China; ^3^Department of Neurology, Xiangya Hospital, Central South University, Changsha, China

**Keywords:** Charcot-Marie-Tooth diseases, concomitant variants, intrafamilial clinical heterogeneity, double trouble, genetic modifier

## Abstract

**Background and Aims:**

Charcot-Marie-Tooth (CMT) disease is a clinically and genetically heterogeneous group of inherited peripheral neuropathies. The wide phenotypic variability may not be completely explained by a single mutation.

**Aims and Methods:**

To explore the existence of concomitant variants in CMT, we enrolled 189 patients and performed molecular diagnosis by application of next-generation sequencing combined with multiplex ligation-dependent probe amplification. We conducted a retrospective analysis of patients harboring coinherited variants in different genes.

**Results:**

Four families were confirmed to possess variants in two genes, accounting for 2.1% (4/189) of the total in our cohort. One CMT1 patient with *PMP22* duplication and *MPZ* variant (c.286A>C, p.K96Q) exhibited moderate neuropathy with infantile onset, while her father possessing *MPZ* variant was mildly affected with adolescence onset. A CMT2 patient with heterozygous variants in *MFN2* (c.613_622delGTCACCACAG, p.V205Sfs^*^26) and *GDAP1* (c.713G>T, p.W238L) exhibited childhood onset mild phenotype, while his mother with *MFN2* variant developed bilateral *pes cavus* only. A CMT2 patient with heterozygous variants in *MFN2* (c.839G>A, p.R280H) and *GDAP1* (c.3G>T, p.M1?) presented infantile onset and rapid progression, while her father with *MFN2* variant presented with absence of deep tendon reflexes. One sporadic CMT2 patient with early onset was confirmed harboring *de novo MFN2* variant (c.1835C>T, p.S612F) and heterozygous *GDAP1* variant (c.767A>G, p.H256R).

**Conclusion:**

Our results suggest that the possibility of concomitant variants was not uncommon and should be considered when significant intrafamilial clinical heterogeneity is observed.

## Introduction

Charcot-Marie-Tooth (CMT) disease is the most common inherited neuropathy with an estimated prevalence of 1 in 2,500 (1). It is a clinically and genetically heterogeneous group of disorders that is characterized by progressive weakness and atrophy of the extremities and loss of sensory function (2). Neurophysiological findings in median nerve differentiate CMT into two groups: demyelinating CMT (CMT1) with slow motor nerve conduction velocity (MNCV) (<38 m/s) and axonal CMT (CMT2) with normal or a slight reduction of MNCV (≥ 38 m/s) (2). It is generally accepted that CMT is a monogenic condition. Up till now, more than 100 causative genes have been reported as causal for CMT (http://neuromuscular.wustl.edu/time/hmsn.html), among which *PMP22* and *MFN2* are the most common causative genes for CMT1 and CMT2, respectively (3). Of note, patients with *PMP22* duplication or *MFN2* mutation, such as p.I126S and p.R94W, have a heterogeneous clinical presentation in terms of age at onset, disease severity, and clinical progression (4, 5). Thus, a single mutation may not completely explain the intrafamilial heterogeneity of CMT. Next-generation sequencing (NGS) affords opportunities to detect the co-occurrence of variants in dual or multiple genes, providing insights into the wide phenotypic variability of the disease.

In this study, we screened 189 Chinese CMT families by using multiplex ligation probe amplification (MLPA) combined with NGS technologies. We are further reporting families with significant intrafamilial phenotypic heterogeneity in the presence of concomitant heterozygous *MFN2* and *GDAP1* variants and a *PMP22* duplication combined with an *MPZ* variant.

## Materials and Methods

### Patients and Clinical Analysis

We recruited 189 unrelated Chinese CMT families from the outpatient neurology clinic of the Third Xiangya Hospital from 2016 to 2020. All the patients were diagnosed by two experienced neurologists according to the CMT diagnostic criteria formulated by the European CMT Consortium (6). Electrophysiological examinations were performed on probands and available family members. Patients were classified into CMT1 (median MNCV <38 m/s) and CMT2 (median MNCV≥38 m/s) subtype accordingly (2). Disease severity was evaluated with the application of the CMT neuropathy score (CMTNS) (7). This study was approved by the Ethics Committee of the Third Xiangya Hospital of Central South University. Written informed consent was obtained from all the participants.

### Genetic Analysis

Genomic DNA was isolated from peripheral blood obtained from all participants using standard Phenol-Chloroform procedures. We first utilized the application of MLPA (P033 kit, MRC Holland, the Netherlands) for the detection of *PMP22* duplication in patients with CMT1. The target NGS was a well-suited and cost-effective strategy for efficient molecular diagnosis of CMT (8–11). Thus, inherited peripheral neuropathy multigene panel (Supplementary Table 1) sequencing was further applied in patients with CMT1 who failed to achieve molecular diagnosis and in patients with CMT2. For patients with concomitant variants, whole-exome sequencing (WES) was carried out to exclude other potential genetic variants. The sample was captured by SureSelect Human All ExonV5 Kit (Agilent). Genomic DNA sequencing was performed on the IlluminaHiseq 2500 platform (San Diego, CA, USA).

### Data Analysis for the Determination of Pathogenic Mutations

All the variants were filtered against the following population database: Genome Aggregation Database (gnomAD), 1,000 Genome project (1,000 genomes), and dbSNP129. *In silico* analyses were performed by Mutation Taster, PolyPhen-2, SIFT, and CADD (Combined Annotation Dependent Depletion) to predict the biological relevance of the amino acid changes and phylogenetic conservation of the mutation sites. Cosegregation analysis was performed utilizing Sanger sequencing to verify the variants. All variants were interpreted according to the American College of Medical Genetics and Genomics (ACMG) standards and guidelines (12).

## Results

### Genetic Findings and Analysis

**Figure 1:**
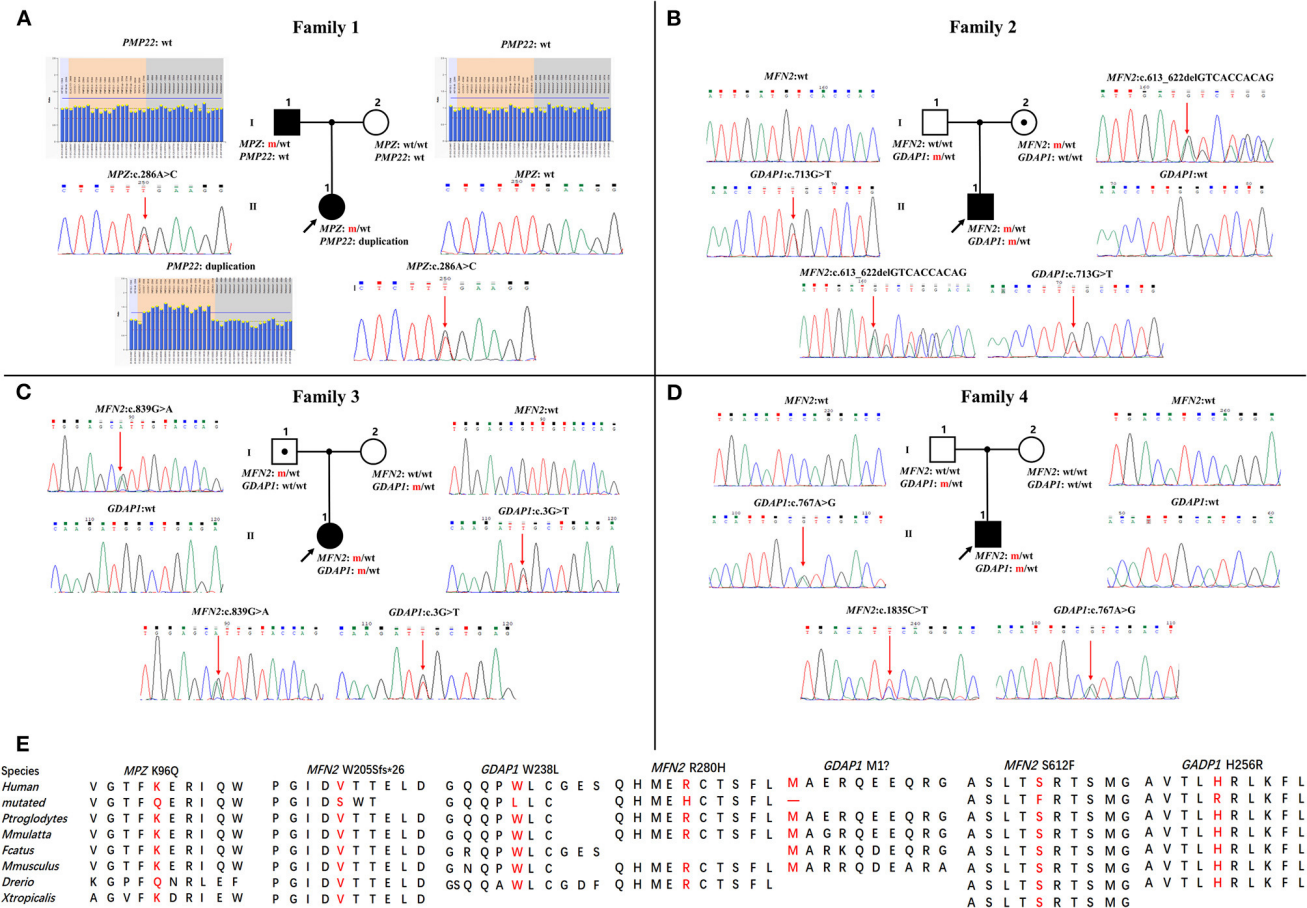
Pedigrees and genomic sequencing results are shown for 4 families with concomitant variants. **(A)** The proband in family 1 has a *PMP22* duplication and an *MPZ* c.286A>C (p.K96Q) variant. **(B)** The proband in family 2 has a combination of heterozygous variants in *MFN2* (c.613_622delGTCACCACAG, p.V205Sfs^*^26) and *GDAP1* (c.713G>T, p.W238L). **(C)** The proband in family 3 possessing heterozygous variants in *MFN2* (c.839G>A, p.R280H) and *GDAP1* (c.3G>T, p.M1?). **(D)** The proband in family 4 with *de novo* heterozygous *MFN2* c.1835 C>T (p.S612F) variant and heterozygous *GDAP1* c.767A>G (p.H256R) variant. Clinical status: open symbols, unaffected family members; filled symbol, patients; black dot in open symbols, subclinical subjects with electromyographic or clinical examination perturbation; the black arrow, proband; wt, wild-type alleles; m, mutated alleles; the red arrow, mutated sites. **(E)** Conservation of amino acids at mutation sites in the different species.

Four families were confirmed to possess variants in two distinct CMT genes. One CMT1 family (F1) harbored a heterozygous *PMP22* duplication and an *MPZ* variant (c.286A>C, p.K96Q) (Figure 1A). Three families with CMT2 carried simultaneous heterozygous variants in *MFN2* and *GDAP1*, among which F2 possessed variants c.613_622delGTCACCACAG (p.V205Sfs^*^26) in *MFN2* and c.713G>T (p.W238L) in *GDAP1*, F3 harbored variants c.839G>A (p.R280H) in *MFN2* and c.3G>T (p.M1?) in *GDAP1*, and F4 with *de novo* c.1835 C>T (p.S612F) variant in *MFN2* and c.767 A>G (p.H256R) variant in *GDAP1* (Figures 1B–D). All the variants were absent or very rare in gnomAD, 1000 Genomes and dbSNP129 (Table 1), were predicted to be damaging by utilizing the application of bioinformatics tool (Table 1), and were well conserved among different species (Figure 1E). We classified them according to the ACMG standards and guidelines and illustrated the results of *in silico* analysis and predicted the pathogenicity of these variants in Table 1. WES was further performed in two families with CMT2 (F2 and F3), and no other potential disease-causing variants were identified.

**Table 1:** Bioinformatics analysis and ACMG classification of the variants detected in 4 families.

				**Population databases**			***In silico*** **analysis**				
**Family**	**Gene**	**Nucleotide changes**	**Amino acid changes**	**gnomAD**	**1000G**	**DbSNP**	**Mutation taster**	**Polyphen2**	**SIFT**	**CADD**	**ACMG classification**
F1	*PMP22*	17p duplication	-	NF	NF	NF	N/A	N/A	N/A	N/A	Pathogenic
	*MPZ*	c.286A>C	p.K96Q	NF	NF	NF	D	B	T	D	Pathogenic (PS1, PM1, PM2, PP1, PP4)
F2	*MFN2*	c.613_622del GTCACCACAG	p.V205Sfs^*^26	NF	NF	NF	D	N/A	N/A	D	Likely pathogenic (PM1, PM2, PM4, PP1, PP3)
	*GDAP1*	c.713G>T	p.W238L	NF	NF	NF	D	D	D	D	VUS
F3	*MFN2*	c.839G>A	p.R280H	NF	NF	rs28940294	D	D	D	D	Pathogenic (PS1, PM1, PM2, PP1, PP3, PP4)
	*GDAP1*	c.3G>T	p.M1?	NF	NF	NF	D	B	D	D	VUS
F4	*MFN2*	c.1835C>T	p.S612F	4.061e-06	NF	rs755299545	D	D	D	D	Likely pathogenic (PS2, PM5, PP3, PP4)
	*GDAP1*	c.767A>G	p.H256R	0.0002	NF	rs1476856429	D	D	D	D	VUS

*gnomAD, Genome Aggregation Database; 1000G, 1000 Genome project; SIFT, Sorts Intolerant From Tolerant; CADD, Combined Annotation Dependent Depletion NF, Not found; N/A, Not appreciable; D, Disease-causing/Damaging; B, Benign; T, Tolerable; ACMG, The American College of Medical Genetics and Genomics; VUS, Variants of uncertain significance*.

We further made frequency comparisons of *GDAP1* variants between patients with CMT and controls (gnomAD) using Pearson chi-squared test. In our cohort, the c.713G>T variant was identified in 1/189 patients (*T* = 1/378), the c.3G>T variant was identified in 2/189 patients (*T* = 2/378), and the c.767A>G variant was identified in 4/189 patients (*G* = 4/378). These three *GDAP1* variants are more frequent in patients with CMT than in controls (*p* < 0.0001).

### Clinical Features of Four Families With Concomitant Variants

F1. The proband, a 6-year-old girl, harboring *PMP22* duplication and *MPZ* variant c.286A>C (p.K96Q) presented with delayed motor milestones and walked independently until 3 years of age. Subsequently, she developed frequent falls and had difficulty walking upstairs or downstairs. The symptoms progressed into her upper limbs with difficulty in buttoning at age 6. Neurological examination revealed atrophy and weakness of the distal upper (scored 3/5) and lower extremities (scored 3/5) and decreased superficial sensations under the ankle. Tendon reflexes were absent in the upper and lower limbs. She presented with bilateral *pes cavus* deformity and walked with a steppage gait. Electrophysiological studies revealed that the amplitudes of compound muscle action potentials (CMAPs) and sensory nerve action potentials (SNAPs) were absent in both the upper and lower limbs. The CMTNS was 15. Her father, a 28-year-old male, with a single *MPZ* variant, noticed his high-arched feet at age 15. He was presented with the reduced athletic ability (running and jumping) compared with his peers and was unable to maintain balance during squatting at age 16. The weakness was gradually progressive without the involvement of the upper extremities. Neurological examination revealed the distal weakness of his lower limbs (scored 4/5), normal sensations, bilateral *pes cavus*, and steppage gait. Tendon reflexes were absent in the lower limbs and reduced in the bilateral upper limbs. Electrophysiological studies revealed that the CMAPs amplitudes were decreased in the upper limbs and absent in the lower limbs and the SNAPs amplitudes were absent in the upper and lower limbs. His CMTNS was 6.

F2. The proband, a 22-year-old male, harboring heterozygous variants in *MFN2* (c.613_622delGTCACCACAG, p.V205Sfs^*^26) and *GDAP1* (c.713G>T, p.W238L) presented with an inability to stand on his heels since age 5. The patient noticed atrophy of the distal lower limbs at 14 years of age and had difficulty in climbing stairs at age 20. Neurological examination showed symmetrical muscle weakness and atrophy in the distal upper (scored 4/5) and lower limbs (scored 0/5), normal sensations, absent knee jerks, and ankle reflexes, *pes cavus*, and steppage gait. Electrophysiological studies revealed decreased amplitudes of CMAPs and SNAPs in the upper and lower limbs. The CMTNS was 9. His mother harboring the same *MFN2* variants was subclinical with *pes cavus* as the only clinical presentation.

F3. The proband with heterozygous variants in *MFN2* (c.839G>A, p.R280H) and *GDAP1* (c.3G>T, p.M1?) was firstly evaluated at the age of 3. She had delayed motor milestones, and frequently sprained her ankle while walking at age 2. On examination, she had symmetrical muscle weakness and atrophy in the distal upper (scored 3/5) and lower limbs (scored 0/5), normal sensations of all modalities, absent ankle reflexes, normal knee jerks, bilateral *pes cavus*, and steppage gait. Electrophysiological studies revealed that the amplitudes of CMAPs were normal in upper limbs and reduced in lower limbs, while the SNAPs amplitudes were decreased in both the upper and lower limbs. The CMTNS was 16. His father carrying a heterozygous variant in *MFN2* (c.839G>A, p.R280H) had subclinical neuropathy with diminished tendon reflexes in the upper and lower limbs as the only clinical sign. Electrophysiological examination revealed slightly decreased CMAP amplitudes in the tibial never and decreased SNAP amplitudes in the upper and lower limbs.

F4. The sporadic patient, a 29-year-old male, harboring heterozygous *de novo MFN2* (c.1835C>T, p.S612F) variant and heterozygous *GDAP1* (c.767A>G, p.H256R) variant noticed foot drop at age 4 and distal atrophy of the calves muscle at age 5. He developed bilateral weakness and atrophy of intrinsic hand muscle at age 7. Neurological examination at age 29 revealed severe atrophy of the distal upper and lower extremities, with reduced strength in foot dorsiflexion (scored 2/5), foot plantarflexion (scored 2/5), and finger abduction (scored 4/5). The sensory examinations were normal. Tendon reflexes were absent in the lower limbs and reduced in the upper limbs. He presented with bilateral *pes cavus* deformity and steppage gait. Electrophysiological studies showed that the amplitudes of CMAPs and SNAPs were reduced in the upper limbs and absent in the lower limbs. The CMTNS was 12. His father and mother were healthy.

Detailed clinical features of the 4 families are summarized in Table 2. Detailed nerve conduction studies are summarized in Supplementary Table 2.

**Table 2:** Clinical features of four families with concomitant variants in this study.

**Family**	**Individual**	**Variants**	**Age at onset/**	**Initial**	**Muscle atrophy**	**Distal muscle**	**Reflexes**		**Pes**	**Sensory**	**CMTNS**
			**exam (years)**	**symptoms**	**(UL/LL)^†^**	**weakness (UL/LL)^‡^**	**(UL/LL)^§^**		**cavus**	**findings (UL/LL)^¶^**	
F1	I-1	*MPZ* p.K96Q	15/28	*Pes cavus*	–/–	5/4	+/–	Steppage	Yes	– / –	6
	II-1 (proband)	*PMP22* duplication + *MPZ* p.K96Q	2/6	Delayed motor milestones	+/+	3/3	–/–	Steppage	Yes	– /– –	15
F2	I-2	*MFN2* p.V205Sfs^*^26	–/46	*Pes cavus*	–/–	5/5	++/++	Normal	Yes	– / –	0
	II-1 (proband)	*MFN2* p.V205Sfs^*^26 + *GDAP1* p.W238L	5/22	Inability to stand on his heels	++/++	4/0	++/–	Steppage	Yes	– / –	9
F3	I-1	*MFN2* p.R280H	–/30	–	–/–	5/5	++/–	Normal	No	– / –	0
	II-1 (proband)	*MFN2* p.R280H + *GDAP1* p.M1I	2/4	Delayed motor milestones	–/++	3/0	++/–	Steppage	Yes	– / –	16
F4	II-1	*MFN2* p.S612F + *GDAP1* p.H256R	4/29	Foot drop	+++/+++	4/2	+/–	Steppage	Yes	– / –	12

† *Muscle atrophy: –: no atrophy; +: mild atrophy; ++: moderate atrophy; +++: severe atrophy (involved in proximal muscle)*.

‡ *Muscle weakness the myodynamia of distal limbs was assessed based on Medical Research Council (MRC) grade 0–5*.

§ *Tendon reflexes: –: loss; +: reduced; ++: normal; +++: brisk; ++++: hyperreflexia*.

¶ *Sensory findings: – –: hypalgesia; –: normal sense; +: hyperpathia*.

*UL, upper limbs; LL, lower limbs; CMTNS, Charcot-Marie-Tooth disease neuropathy score*.

## Discussion

**Figure 2:**
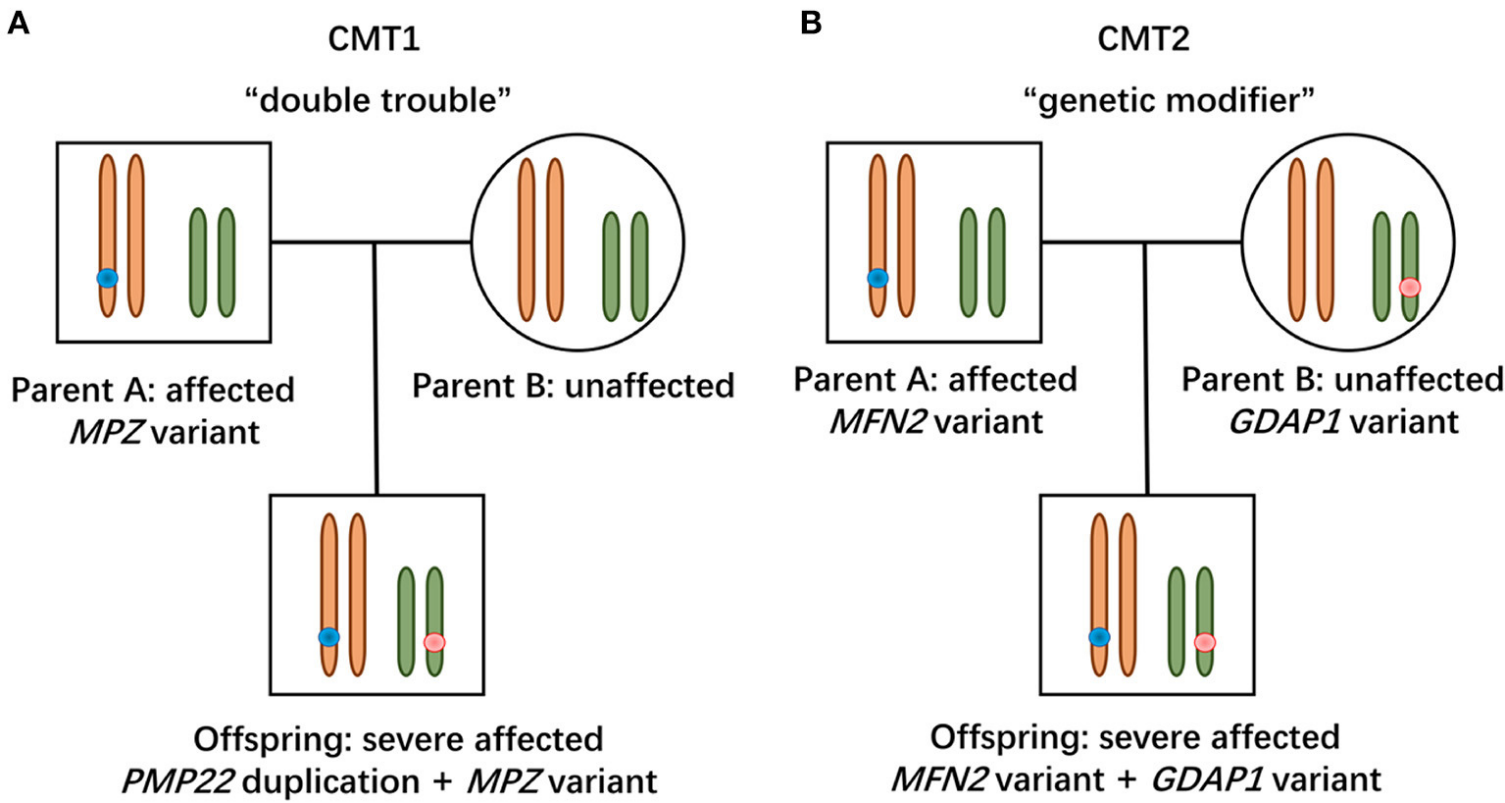
Modes of concomitant variants in CMT. **(A)** Patient with CMT1 harboring *de novo PMP22* duplication and inherited *MPZ* variant presented with a severe phenotype, suggesting a “double trouble” effect. **(B)** CMT2 patients possessing concomitant variants in *MFN2* and *GDAP1* were severely affected, indicating that the heterozygous state of *GADP1* variants serves as a “genetic modifier.”

In this study, the patient with CMT1 harboring a *de novo PMP22* duplication and an inherited *MPZ* variant presented with an earlier disease onset and a severer phenotype than her father harboring only the *MPZ* variant (Figure 1A). Each variant was sufficient to cause disease, suggesting a cumulative “double trouble” effect of these two concomitant variants (Figure 2A). MPZ is the major structural protein of peripheral myelin expressed by Schwann cells (more than 50%) (13). Mutation in *MPZ* was associated with unfolded protein response activation and protein aggregates, leading to the apoptosis and demyelination of the Schwann cell and altered axonal interaction (13, 14). PMP22 is also highly expressed in myelinating Schwann cell (2–5%) and directly contribute to myelin organization (15). *PMP22* duplication disrupts the development and maintenance of normal myelin in the Schwann cell (16). Thus, simultaneous variants in these two myelin-related genes might exert double deleterious effects in Schwann cells, which finally leads to more severe defects in the myelination of axons. Of interest, the co-occurrence of *PMP22* duplication and variant in another CMT gene (*GJB1, LITAF*) had also been reported (17, 18). Considering *PMP22* duplication as the most common cause of CMT1 patients (approximately 70%) (3, 19, 20), one of the changes observed in cases with CMT1 with variants in two genes is usually the *PMP22* duplication.

Two patients with concomitant variants in *MFN2* and *GDAP1* were severer affected than their parents harboring a single *MFN2* variant (Figures 1B,C). Heterozygous *MFN2* variants were the primary mutations that could establish the diagnosis. The variant c.3G>T in *GDAP1* changed the initiation codon, which has been reported in previous literature (21–23). The *GDAP1* c.713G>T missense change occurred at the same position (p.W238) as another pathogenic variant observed (21, 24). The bioinformatic analysis supported the deleterious effect of both variants. Of note, WES ruled out other candidate genetic variants that could be involved in phenotypic expression in these families. Taken together, the heterozygous state of *GADP1* variants could increase the disease severity in association with the inherited *MFN2* variant, indicating their role of “genetic modifier” (Figure 2B). Another patient with CMT2 with *de novo MFN2* variant and inherited heterozygous *GDAP1* variant (Figure 1D) presented with early disease onset and moderate phenotype. The variant c.767A>G in *GDAP1* is widely recognized as causative of autosomal recessive (AR) CMT (25–27) and might also modulate phenotypic expression in this case. MFN2 and GDAP1 serve as a protein partners in regulating mitochondrial dynamics (MFN2 for fusion, GDAP1 for fission) and are involved in the same mitochondrial function such as energy coupling (28, 29). Mutant GDAP1 exerts a loss of function mechanism and aggravate mitochondrial damage (dynamics dysfunction and energy deficit) caused by *MFN2* mutation, which could explain the exacerbation of the CMT2A phenotype. The deficits in mitochondrial function might be their underlying mechanism which still needs to be further studied. *MFN2* and *GDAP1* are among the more frequent causative genes of CMT2, and this might be the reason why concomitant variants in these two genes have been repeatedly encountered in patients with variants in two CMT2 genes. Of interest, the frameshift p.V205Sfs^*^26 variant in *MFN2* associated with subclinical neuropathy in our study suggested that *MFN2* is not sensitive to haploinsufficiency. Previous reports have described patients with heterozygous state of *MFN2* variants (p.E308X, p.V160fs^*^26 and del ex7-8) developing a subclinical phenotype or asymptomatic presentation (30, 31), indicating that null variants in *MFN2* might be associated with a minimal phenotype.

This study identified a 2.1% (4/189) prevalence of concomitant variants in our cohort. The concomitant variants in families with CMT have been documented in several studies. Recently, a Japanese cohort study identified 5 out of 1,005 families with CMT harboring coinherited variants, accounting for 0.5% (5/1,005) of the total, in which the “double trouble” effect of concomitant variants were the underlying causes (32). Compared with the “genetic modifier” effect of *GDAP1* variants observed in this study, the coexistence of homozygous AR-CMT2K (p.Q163X) or heterozygous autosomal dominant-CMT2K (p.H123R, p.E222K, and p.R120W) variant in *GDAP1* and heterozygous variant in *MFN2* had been reported associating with a more severe phenotype, suggesting the “double trouble” effect of concomitant *MFN2* and *GDAP1* variants (28, 30, 33, 34). Moreover, concomitant variants were also related to true digenic inheritance (DI) that has been defined as the coinheritance of two nonallelic mutations, both are indispensable to establish the diagnosis (35). The existence of true DI was reported in a family with CMT with heterozygous *MFN2* p.L741V and *GDAP1* p.Q163^*^ variants (36). These results suggest that the occurrence of concomitant variants was not uncommon in CMT.

There was an important limitation to this study. Because we conducted a retrospective analysis of target NGS data, genetic variants in non-CMT-causing genes could not be detected. WES (or whole-genome sequencing [WGS]) would detect all possible genetic modifiers. The application of WES (WGS) in all the families with phenotypic variabilities could provide further insights into the clinical and genetic heterogeneity of CMT.

In summary, the coexistence of variants in *PMP22*/*MPZ* and *MFN2*/*GDAP1* related to more severe phenotypes accounted for 2.1% of patients with CMT in our cohort. Cumulative deficits on myelination or mitochondrial function might be their underlying mechanism. The possibility of the coexistence of variants in distinct causative genes should be taken into consideration when significant clinical heterogeneity is observed.

## Data Availability Statement

The data presented in this study were deposited in Figshare open access repository doi: 10.6084/m9.figshare.17192927. Further inquiries can be directed to the corresponding author.

## Ethics Statement

The studies involving human participants were reviewed and approved by the Ethics Committee of the Third Xiangya Hospital of Central South University. Written informed consent to participate in this study was provided by the participants' legal guardian/next of kin.

## Author Contributions

RZ designed and conceptualized study. YX, ZL, XL, LL, BW, WC, JG, LS, and RZ contributed patient material and clinical data. SH and HZ contributed acquisition of neurophysiological data. ZL and ZH interpreted the genetic data. YX provide the first draft of the manuscript. RZ and BT revised the manuscript. All authors contributed to the article and approved the submitted version.

## Funding

This study is funded by the National Natural Science Foundation of China (81771366 and 82001338) and the China International Medical Foundation (CIMF-Z-2016-20-1801).

## Conflict of Interest

The authors declare that the research was conducted in the absence of any commercial or financial relationships that could be construed as a potential conflict of interest.

## Publisher's Note

All claims expressed in this article are solely those of the authors and do not necessarily represent those of their affiliated organizations, or those of the publisher, the editors and the reviewers. Any product that may be evaluated in this article, or claim that may be made by its manufacturer, is not guaranteed or endorsed by the publisher.
