# Supplementary material for: One PMP22/MPZ and Three MFN2/GDAP1 Concomitant Variants Occurred in a Cohort of 189 Chinese Charcot-Marie-Tooth Families
Source: Front Neurol. 2022 Jan 28;12:736704. doi: 10.3389/fneur.2021.736704 (PMC8831722; doi:10.3389/fneur.2021.736704)
Supplement: Supplementary file 1 [file Table_1.DOCX]

**Supplementary Material**

**Supplementary Table 1. The gene list of inherited peripheral neuropathy multi-gene panels**

| *AAAS* | *ARHGEF10* | *AARS* | *ABHD12* | *AIFM1* | *ATP7A* | *ARSA* | *ASAH* | *ATL1* | *ATSV* | *BSCL2* | *CCT5* | *COX6A1* | *CTDP1* |
| --- | --- | --- | --- | --- | --- | --- | --- | --- | --- | --- | --- | --- | --- |
| *CUL4B* | *DCAF8* | *DCTN1* | *DGAT2* | *DHH* | *DNM2* | *DNMT1* | *DNAJB2* | *DNAJC3* | *DHTKD* | *DRP2* | *DYNC1H1* | *EGR2* | *EMILIN1* |
| *EXOSC3* | *FAM134B* | *FBLN5* | *FBXO38* | *FGD4* | *FIG4* | *FLVCR1* | *GALC* | *GARS* | *GDAP1* | *GJB1* | *Gigaxonin* | *GJB3* | *GNB4* |
| *HARS* | *HEXA* | *HINT1* | *HOXD10* | *HK1* | *HSJ1* | *HSPB1* | *HSPB3* | *HSPB8* | *IFRD1* | *IKBKAP* | *IGHMBP2* | *INF2* | *KARS* |
| *KCC3* | *KIF1A* | *KIF1B* | *KIF5A* | *KLHL9* | *LMNA* | *LAS1L* | *LITAF* | *LRSAM1* | *MARS* | *MED25* | *MFN2* | *MME* | *MORC2* |
| *MTMR2* | *MT-ATP6* | *MYH14* | *NAGLU* | *NEFH* | *NEFL* | *NDRG1* | *NGF* | *NTRK1* | *MPZ* | *PDK3* | *Periaxin* | *PEX1* | *PEX7* |
| *PHYH* | *PLEKHG5* | *PLA2G6* | *PMM2* | *PMP2* | *PMP22* | *PNKP* | *PRNP* | *PRPS1* | *RAB7* | *RBM28* | *REEP1* | *RNF170* | *SBF1* |
| *SBF2* | *SIGMAR1* | *SCYL1* | *SH3TC2* | *SCP2* | *SMN1* | *SPTLC1* | *SLC5A7* | *SLC12A6* | *SOX10* | *SPG11* | *SLC25A46* | *SPTLC2* | *SPTLC3* |
| *SURF1* | *TDP1* | *TFG* | *TRIM2* | *TRPV4* | *TUBB3* | *UBA1* | *VCP* | *VRK1* | *YARS* |  |  |  |  |

**Supplementary Table 2. Nerve conduction studies of patients in 4 families**

|  | Family 1 | | Family 2 | | Family 3 | | Family 4 | Normal value |
| --- | --- | --- | --- | --- | --- | --- | --- | --- |
|  | I-1 | II-1 | I-2 | II-1 | I-1 | II-1 | II-1 |  |
| Motor nerves  NCV(m/s) /CMAP(mV) |  |  |  |  |  |  |  |  |
| Median nerve | 20/1.3 | A | 59.4/5.4 | 51/4.0 | 59.3/11.5 | 51/6.4 | 49.5/0.1 | >50/>5.0 |
| Ulnar nerve | 23/7.3 | A | 63.5/7.1 | 51/7.0 | 59.0/8.8 | 55.7/6.3 | 32.1/0.2 | >50/>7.0 |
| Peroneal nerve | A | 3.3/0.4 | 52.9/3.3 | 26.9/3.0 | 40.8/7.8 | 33.8/0.2 | A | >40/>2.5 |
| Tibial nerve | A | A | 50.7/9.6 | 22.2/2.0 | 45.3/4.1 | 36.6/1.1 | A | >37/>4.8 |
| Sensory nerves  NCV(m/s) /SNAP(uV) |  |  |  |  |  |  |  |  |
| Median nerve | A | A | ND | 54/1.1 | 63.7/16 | 35.4/1.1 | 42.6/8.2 | >44/>20 |
| Ulnar nerve | A | A | ND | 47.4/1.0 | 69.5/12 | 37.5/1.0 | 37/1.7 | >44/>20 |
| Peroneal nerve | A | A | 67.3/30 | 17.5/0.5 | 41.9/2.3 | 16.9/0.4 | A | >41/>6.0 |
| Sural nerve | ND | A | ND | ND | 44.7/3.2 | 12.5/0.4 | A | >36/>10 |

NCV: nerve conduction velocity; CMAP: compound muscle action potential; SNAP: sensory nerve action potential; A: absent evoked response; ND: not done.
